# Complete Genome Sequence of the *Streptococcus* sp. Strain VT 162, Isolated from the Saliva of Pediatric Oncohematology Patients

**DOI:** 10.1128/genomeA.00647-14

**Published:** 2014-07-10

**Authors:** Maria F. Vecherkovskaya, George V. Tetz, Victor V. Tetz

**Affiliations:** Institute of Human Microbiology LLC, Department of Microbiology, Virology and Immunology, First State I. P. Pavlov Medical University, Saint Petersburg, Russia

## Abstract

*Streptococcus* sp. strain VT 162 was isolated from the saliva of pediatric oncohematology patients. Its full genome is 2,045,418 bp. The availability of this genome will provide insights into the composition of microbial flora among pediatric oncohematology patients and the host interaction and pathogenicity of this species.

## GENOME ANNOUNCEMENT

*Streptococcus* species are Gram-positive facultatively anaerobic cocci. Streptococci are widely present in the oral cavity and are established part of commensal flora (1). In pediatric cancer patients, and in oncohematology patients in particular, streptococci are known to cause a wide variety of conditions ranging from local (caries, mucositis, stomatitis, and gingivitis) (2, 3) to generalized (septicemia and sepsis) complications (4, 5).

*Streptococcus* sp. strain VT162 was isolated from the saliva of several patients with oncohematological malignancies in remission during their follow-up visits. Biochemical and matrix-assisted laser desorption ionization–time of flight mass spectrometry (MALDI-TOF MS) identifications gave divergent low-discrimination results. Phylogenetic analysis of the 16S rRNA gene sequence had placed this isolate among streptococci, but identification to the species level was still inconclusive. Thus, whole-genome sequencing was performed.

The *Streptococcus* sp. strain VT162 was sequenced using the Illumina HiSeq 2500 system sequencing technology (Illumina GA IIx; Illumina, CA). The library preparation, sequencing reaction, and the sequencing run were carried out according to Illumina’s instructions. A total of 16.79 million high-quality 51-bp single-end reads were produced, resulting in approximate coverage of 345×. Assembly was performed using Velvet assembler version 1.2.10 (6) and resulted in 340 contigs. The contig *N*_50_ is 42,108 bp, and the largest assembled contig is 115,694 bp. To produce the chromosome assembly, the contigs were oriented and ordered by alignment with the genome of *Streptococcus oralis* Uo5 (7) using the Mauve aligner version 2.3.1 (8).

Annotation was added by the NCBI Prokaryotic Genome Annotation Pipeline (2013 release). The VT 162 chromosome is 2,045,418 bp in length, with a G+C content of 41.1%. There are 1,935 predicted protein-coding sequences (CDSs), with an average length of 909 bp, and 34 tRNAs, 3 rRNAs, and 1 noncoding RNA (ncRNA) gene were identified.

The genome contains genes for several competence proteins and multidrug resistance transporters of the ABC, MATE, MFS, and DMT families, the *vanZ* gene that confers resistance to teicoplanin, and genes for hemolysin III and capsular and capsid proteins.

The average nucleotide identity (ANI) (9) demonstrates that *Streptococcus* sp. VT 162 differs significantly from the mitis phylogenetic group of streptococcus, showing ANI values of <95% compared to the genomes of the closest species, such as *S. oralis* Uo5 (7).

The complete genome sequence of *Streptococcus* sp. VT 162 will help in establishing a new species of *Streptococcus* and will provide insights into the composition of microbial flora among pediatric oncohematology patients, as well as the host interaction and pathogenicity of this species.

### Nucleotide sequence accession number.

The complete genome sequence has been deposited in the NCBI database under accession no. CP007628.
